# Unraveling the reaction mechanisms for furfural electroreduction on copper†

**DOI:** 10.1039/d3ey00040k

**Published:** 2023-04-28

**Authors:** Sihang Liu, Zamaan Mukadam, Soren B. Scott, Saurav Ch. Sarma, Maria-Magdalena Titirici, Karen Chan, Nitish Govindarajan, Ifan E. L. Stephens, Georg Kastlunger

**Affiliations:** a Department of Physics, Catalysis Theory Center, Technical University of Denmark (DTU) 2800 Kgs. Lyngby Denmark geokast@dtu.dk; b Department of Materials, Royal School of Mines, Imperial College London London SW27 AZ England UK i.stephens@imperial.ac.uk; c Department of Chemical Engineering, Imperial College London London SW7 2AZ England UK; d Advanced Institute for Materials Research (WPI-AIMR), Tohoku University Sendai Miyagi 980-8577 Japan; e Materials Science Division, Lawrence Livermore National Laboratory Livermore California 94550 USA govindarajan1@llnl.gov

## Abstract

Electrochemical routes for the valorization of biomass-derived feedstock molecules offer sustainable pathways to produce chemicals and fuels. However, the underlying reaction mechanisms for their electrochemical conversion remain elusive. In particular, the exact role of proton–electron coupled transfer and electrocatalytic hydrogenation in the reaction mechanisms for biomass electroreduction are disputed. In this work, we study the reaction mechanism underlying the electroreduction of furfural, an important biomass-derived platform chemical, combining grand-canonical (constant-potential) density functional theory-based microkinetic simulations and pH dependent experiments on Cu under acidic conditions. Our simulations indicate the second PCET step in the reaction pathway to be the rate- and selectivity-determining step for the production of the two main products of furfural electroreduction on Cu, *i.e.*, furfuryl alcohol and 2-methyl furan, at moderate overpotentials. We further identify the source of Cu's ability to produce both products with comparable activity in their nearly equal activation energies. Furthermore, our microkinetic simulations suggest that surface hydrogenation steps play a minor role in determining the overall activity of furfural electroreduction compared to PCET steps due to the low steady-state hydrogen coverage predicted under reaction conditions, the high activation barriers for surface hydrogenation and the observed pH dependence of the reaction. As a theoretical guideline, low pH (<1.5) and moderate potential (*ca.* −0.5 V *vs.* SHE) conditions are suggested for selective 2-MF production.

## Abstract 2

Combining theory and experiment, we unveil the mechanisms for furfural electroreduction on copper and provide a rationale to tune reaction conditions to increase activity towards value-added products.

## Graphical abstract

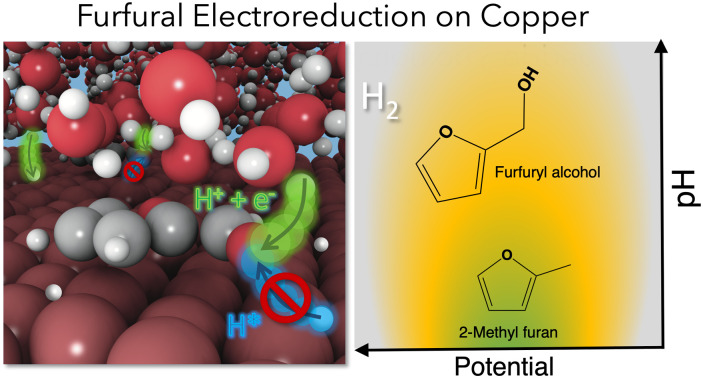

**Broader context**

The electro-valorization of biomass-derived chemicals has the potential to enable the sustainable production of value-added chemicals and biofuels using green electricity and an abundant source of protons. One of the most studied processes in this regard is the electroreduction of furfural, a lignin-derived platform chemical. Copper electrodes have been used to electrocatalytically reduce furfural to both furfuryl alcohol and 2-methyl furan, key precursors of polymers and drop-in jet-fuels, respectively. However, the underlying reaction mechanism remains elusive. As furfural-fed electrolyzers are being developed, optimized, and scaled up, the question arises as to whether and how we can more effectively leverage potential, pH and other electrochemical parameters to upgrade furfural into target products, especially for the production of highly profitable biofuels. An efficient advance of this scope urges us to solve the fundamental mechanistic puzzles within furfural electroreduction.

## 1 Introduction

The electrochemical conversion of biomass-derived feedstocks towards value-added chemicals offers a sustainable route to decarbonizing the chemical industry.^1,2^ Furfural (FCHO, F represents the furan ring and CHO the aldehyde group) is one of the most abundant biomass-derived platform chemicals with an annual production capacity of more than 2 million tons.^3^ It serves as a critical feedstock towards the production of downstream chemicals and fuels such as furfuryl alcohol (FAL), 2-methylfuran (2-MF), tetrahydrofurfuryl alcohol, hydrofuroin and other furanic derivatives.^3,4^ The electrochemical furfural reduction reaction has several advantages over thermal hydrogenation including tunable selectivity *via* applied potential or current, mild reaction conditions, the possibility of coupling to intermittent sources of renewable electricity (green electrons), and the use of water as the hydrogen source. All these aspects can dramatically reduce the carbon footprint of biomass conversion processes. Therefore, electrochemical routes for the reduction of furfural have received increased attention in recent years, although earliest attempts date back to the late 19th century.^5^

**Fig. 1:**
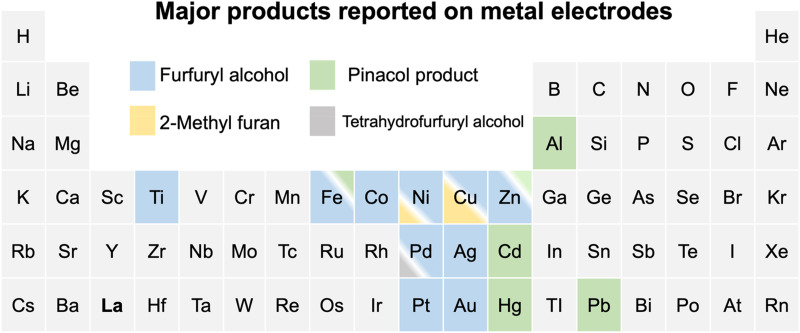
A summary of reported major products of aqueous-phase furfural reduction reaction on metal electrodes at moderate potentials. Note that the ratios of colored regions in the box suggest the relative selectivity under similar reaction conditions and the minor products are omitted. Cu is the only metal that is highly selective towards both furfuryl alcohol and 2-methyl furan at different acidic conditions.^5–14^ The details for reported experiments are summarized in Table S1 (ESI†).

**Scheme 1:**
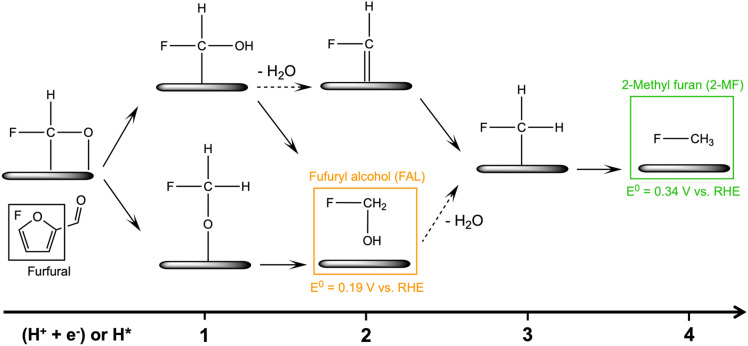
Proposed reaction mechanisms for the furfural electroreduction reaction towards furfuryl alcohol (FAL) and 2-methylfuran (2-MF) on Cu surfaces.^17,18^ (H^+^ + e^−^) and H* denote proton-coupled electron transfer (PCET) and surface hydrogenation (where H* is produced *via* the Volmer reaction), respectively. The solid or dashed arrows represent elementary reduction step without or with H_2_O production.

The past decade has witnessed great progress in exploring furfural reduction over different metal electrodes, and the major products reported in previous studies are summarized in Fig. 1. Furfuryl alcohol and hydrofuroin are the most common products, while 2-methyl furan and tetrahydrofurfuryl alcohol are only selectively produced on limited metals at very acidic conditions.^5–14^ In particular, copper shows potential as a multi-selective electrocatalyst for furfural reduction, as it is able to produce both FAL, a precursor to polymers and resin,^15^ and 2-MF, a well-known alternative drop-in biofuel,^16^ with nearly 100% selectivity at moderate potentials (*ca.* −0.6 V *vs.* RHE).^14^ Possible reaction pathways for furfural electroreduction to FAL and 2-MF on Cu have been proposed by Chadderdon *et al.*^17^ and Shan *et al.*^18^ (*cf.*Scheme 1). Furfural (FCHO*), where * indicates a surface adsorbed species, is first reduced to either FCHOH* or FCH_2_O*, *via* a proton-coupled electron transfer (PCET) or surface hydrogenation of the oxygen or carbon species on FCHO*, respectively. These intermediates are suggested to be further reduced to FAL or 2-MF *via* subsequent PCET (or surface hydrogenation) steps. Alternatively, FAL has also been proposed to be a precursor for 2-MF formation.^18^

Several attempts have been made to narrow down the mechanistic possibilities and identify the rate-determining step(s) (RDS) towards the products of furfural reduction on Cu electrodes.^14,19,20^ For instance, Nilges and Schroder^14^ reported a lower overpotential for furfural reduction compared to HER on Cu electrodes under acidic conditions, indicating the activation barriers towards FAL and/or 2-MF are lower than those for H_2_ production. However, the actual mechanism for furfural reduction was not explored in this experimental study. More recently, May *et al.*^19^ measured partial current densities and the reaction order with respect to furfural for the production of FAL and 2-MF on Cu. Using a simple microkinetic model, they proposed the surface hydrogenation of adsorbed furfural (FCHO*) and C–O bond dissociation as the RDS towards FAL and 2-MF, respectively. We note that May and co-workers did not consider the possibility of PCET based pathways, where the adsorbed furanic intermediates are directly protonated by the solvent (*i.e.*, H_3_O^+^/H_2_O). Finally, Jung *et al.* reported that upon feeding FAL as the reactant for electroreduction on Cu, 2-MF was not detected as a product.^20^ This observation rules out the hypothesis that FAL is a precursor to produce 2-MF.

The competition between proton coupled electron transfer (PCET), and electrochemical catalytic hydrogenation (ECH) in furfural reduction, where surface adsorbed species are hydrogenated by protons from the electrolyte (coupled to electron transfer from the electrode) or surface adsorbed hydrogen (denoted by H*), respectively, is still in disputed. Chadderdon *et al.*^17^ and Liu *et al.*^21^ proposed that ECH-based mechanisms are dominant in furfural reduction towards FAL and 2-MF on Cu electrodes, while an outer-sphere reaction pathway might dominate hydrofuroin production. The researchers used self-assembled monolayers of thiols with varying carbon-chain lengths to coat the Cu or Pb surfaces to reduce surface adsorption. They attributed the resulting decrease in the production of 2-MF (more drastically) and FAL to the reduction in surface hydrogen coverages, leading to a proposed ECH mechanism on Cu. However, the surface poisoning experiments could only confirm the limiting steps for production of 2-MF and FAL are inner-sphere reactions, but do not rule out the pathway *via* direct protonation to adsorbed furanic intermediates (an Eley–Rideal pathway), which was not explicitly discussed in the mechanisms proposed on Cu electrodes.^17^ In addition, their measured kinetic isotope effects lead to the conclusion that furfural reduction on Cu proceeds *via* an ECH mechanism due to a similar KIE behavior to HER. However, inner sphere PCET steps onto furfural adsorbed on the electrode would also lead to a KIE comparable to HER. Thus, we argue that neither surface poisoning or KIE experiments allows us to distinguish between a surface hydrogenation and PCET-based mechanisms. As further evidence for the reaction mechanism, May *et al.* showed that selectivity to 2-MF could be dramatically reduced in favor of FAL with an increase in the electrolyte pH.^22^ This conclusion was further strengthened in a recent study by Xu *et al.* where the authors reported close to 100% selectivity to FAL on a Cu electrode supported on N-doped porous carbon at pH = 13.6.^23^ The strong pH-dependence of the product distribution suggests that the formation of at least one of the two products directly involves protons (or hydroxides) from solution rather than the ECH mechanism exclusively based on the involvement of H*.

Computational studies are few in furfural reduction reaction. Lopez-Ruiz *et al.* presented rate expressions with extreme-scenario assumptions to show that both ECH/PCET based pathways could describe the activity trends for furfural reduction on Cu.^24^ Shan *et al.* calculated the reaction energetics of ECH mechanisms and concluded that the first hydrogenation step and the C–O bond scission are possible rate-limiting steps for the formation of FAL and 2-MF, respectively.^18^ However, previous theoretical studies have either neglected the activation energies associated with PCET based reaction steps in the furfural reduction mechanism or approximated them based on surface hydrogenation barriers that are insensitive to changes in applied potential and pH.

Herein, we combine constant-potential DFT based microkinetic simulations, including both the PCET and ECH pathways, and pH dependent experiments under acidic conditions to study furfural reduction on Cu. The calculated reaction energetics show that although FCH_2_O* is thermodynamically favored over FCHOH*, its formation is kinetically hindered on Cu(111) at relevant potentials. The microkinetic simulations including a degree of rate control analysis indicate that the second PCET step, *i.e.*, the protonation of C *vs.* O in FCHOH* is the rate- and selectivity determining steps at moderate overpotentials towards FAL and 2-MF, respectively. Furthermore, we find that the ECH pathway plays a minor role in furfural reduction due to a combination of the low coverage of H* predicted on Cu terrace sites and the high activation energies associated with the surface hydrogenation steps. We provide further evidence to our mechanistic conclusions by evaluating the potential and pH dependence (*vs.* the reversible hydrogen electrode) of both FAL and 2-MF production, which strongly suggests the involvement of a later PCET step in the RDS for furfural reduction on Cu electrodes. Guidelines for selective 2-MF production are proposed. The mechanistic insights obtained herein shed light on the competing reaction pathways in furfural reduction and provide a framework to understand reaction mechanisms in electrochemical biomass valorization, as well as multi-step electrochemical reactions in general.

## 2 Results and discussion

### 2.1 The formation of FCHOH* is kinetically favored relative to the more thermodynamically stable FCH_2_O*

**Fig. 2:**
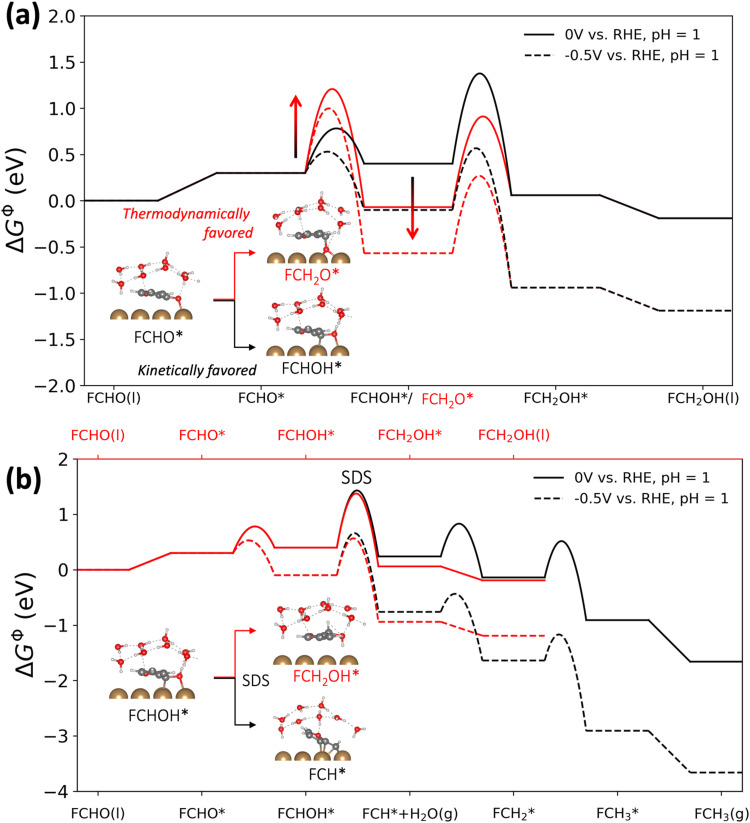
The calculated constant-potential free energy diagrams of furfural reduction on Cu(111) surface. (a) The competition of reaction thermodynamics and kinetics in the PCET steps from furfural (FCHO*) to the adsorbates FCHOH* and FCH_2_O*; (b) the complete free energy profiles to both products: furfuryl alcohol (FCH_2_OH, red) and 2-methyl furane (FCH_3_, black). Color code in the insets: brown-Cu, dark grey-C, red-O, and light grey-H.

The reaction energetics (*i.e.* thermodynamics and activation barriers) were computed using DFT for all (electro-)chemical elementary steps illustrated in Scheme 1 and eqn (1) and (2) on Cu(111) under very acidic conditions (*i.e.* where we assume the major proton donor is H_3_O^+^), employing a grand-canonical framework.^25^ An ice-like water structure was used to represent the solvent structure in our model,^26^ in order to approximate the endothermic solvent effect on the adsorption of larger molecules like furan compounds having a sizeable energetic penalty due to water displacement.^27,28^ A slightly tilted orientation of furfural adsorption on Cu(111) was obtained shown in the inset of Fig. 2(a), in line with a recent molecular dynamics study.^29^ The applied computational model is shown in Fig. S1 (ESI†). We applied the symmetry factor *β* obtained from grand-canonical (GC) constant-potential calculations to quantify the response of the determined electrochemical activation energies to potential, as summarized in Table S2 (ESI†), as described in ref. 30. Note that (1) we do not include the analysis of dimer products in our study, because they are minor outer-sphere products on copper under acidic conditions;^17,21^ (2) cation effects are not considered in this work, as the experiments were carried out in HClO_4_ solutions free of metal cations. However, we note that investigating the effects of cation/anion identity (and microenvironment effects in general) on furfural electroreduction would be an interesting future direction.

1 FCHO(l) + 2(H^+^ + e^−^) ↔ FCH_2_OH(l)

2 FCHO(l) + 4(H^+^ + e^−^) ↔ FCH_3_(g) + H_2_O(l)

We find that the formation of FCH_2_O* is associated with a higher activation barrier than the formation of FCHOH* (*cf.*Fig. 2(a)), even though FCH_2_O* is thermodynamically more stable than FCHOH*. FCHOH* prefers to adsorb in a configuration with the –CHOH group binding at an atop site, while the –CH_2_O moiety in *FCH_2_O binds to a hollow site, resulting in *ca.* 1 eV higher thermodynamic stability relative to FCHOH* (*cf.* inset, Fig. 2(a)). However, we find the activation barrier for the formation of FCH_2_O* *via* a PCET step using H_3_O^+^ as the proton source to be substantially higher than the corresponding PCET barrier for the formation of FCHOH* (0.90 eV *vs.* 0.48 eV at 0 V *vs*. RHE, pH 1 and 0.66 eV *vs.* 0.20 eV at −0.5 V *vs*. RHE, pH 1) in line with the general trend of electrochemical barriers for protonating C and O on metals reported by Patel *et al.*^31^ Furthermore, our simulations indicate that the further protonation of FCH_2_O* is sterically hindered, as it requires the proton from the electrolyte to approach the surface-bound oxygen on the hollow site. Hence, the hydrogenation of FCH_2_O* would prefer H* on the surface as a reactant (*i.e.*, following the ECH pathway), which is shown to be kinetically hindered in the following section.

Fig. 2(b) shows the free energy profiles towards FAL and 2-MF, highlighting the identified selectivity determining step (SDS) which is the protonation of FCHOH* to FCH_2_OH* (FAL pathway) or to FCH* (2-MF pathway). We note that the second PCET step, *i.e.*, the protonation of FCHOH*, is also predicted to have the highest barrier along the reaction pathway at both potentials, hence functioning as both the RDS and SDS. We find that the SDS towards FCH_2_OH* and FCH* displays comparable activation barriers which is in agreement with their comparable activity on Cu towards both FAL and 2-MF in acidic media.^14,17^ In addition, we calculated FCH_2_OH* to FCH_2_* to have a formidably high activation energy of 1.46 eV, which defies the production of 2-MF from adsorbed FAL *via* a PCET pathway.

In order to study the competing hydrogen evolution reaction on Cu(111), we also explicitly calculated the HER energetics under acidic conditions using GC-DFT (*cf.* Fig. S2, ESI†). We identify the Volmer step as the RDS in H_2_ production on Cu(111), with a barrier of *ca.* 1 eV at 0 V *vs.* RHE and pH 1, higher than the activation energies of the limiting PCET steps involved in furfural reduction under the same conditions.

### 2.2 The dominance of furfural reduction at low overpotentials compared to HER strongly indicates the dominance of PCET based pathways

**Fig. 3:**
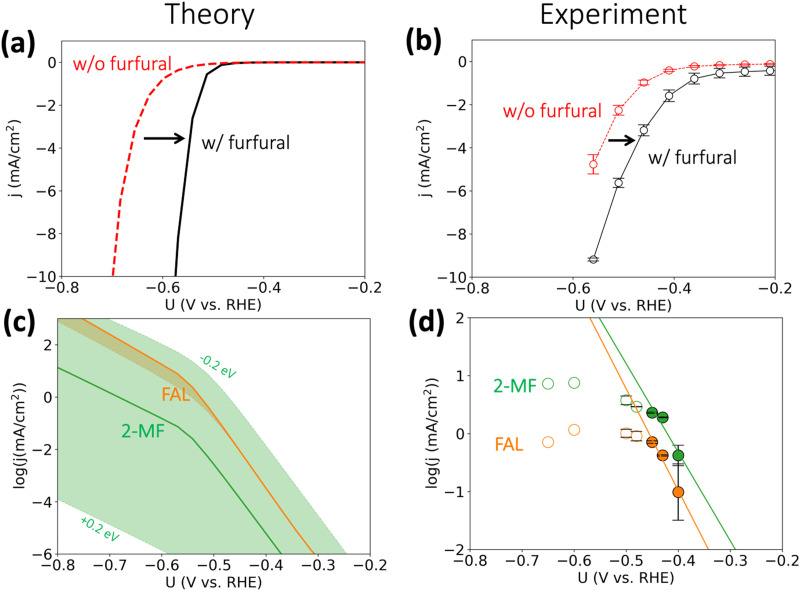
Activity for furfural reduction reaction on Cu. (a and b) Simulated and experimental total polarization curves with furfural reduction (in black) and without furfural (HER, in red); (c) and (d) simulated and experimental partial current densities (on log scale) towards furfuryl alcohol (FAL) and 2-methyl furan (2-MF). Simulated reaction conditions: 100 mM furfural, 300 K, pH = 1. We applied ±0.2 eV error estimates for the barrier of selectivity (rate)-determining step for 2-MF formation *i.e.*, FCHOH* → FCH* to account for the intrinsic uncertainty in DFT calculations^34^ and electrochemical interface simulations, which is represented by the orange and green shade areas in (c). Reaction conditions: 0.1 M HClO_4_ electrolyte (pH 1), 8 mM furfural, constant potential was applied for three hours. Error bars were produced using the results of two separate experiments, where each point warranted a fresh experiment. All experimental potentials were reported with iR corrections. No repeats were performed for −0.60 and −0.65 V *vs.* RHE as mass transport limitations were already reached at these potentials.

On the basis of the calculated reaction energetics, we developed a mean-field microkinetic model to obtain mechanistic insights into acidic furfural reduction on Cu(111). As expected from the discussion in the previous section, cathodic current at potentials more negative than −0.5 V *vs*. RHE on Cu electrode is predicted to dramatically increase when there is furfural in the electrolyte, as shown in Fig. 3(a). This observation is attributed to much higher rates of furfural reduction than hydrogen evolution (*cf.* Fig. S3, ESI†) and is in in qualitative agreement with the experimental current densities obtained under similar conditions (*i.e.*, pH = 1), shown in Fig. 3(b). Notably, the simulated coverage map in Fig. S4 (ESI†) shows that the intermediate FCHOH* and FCH_2_O* already reach moderate coverages at quite low current densities, rationalizing a facile adsorption and first protonation step. This agrees with the finding that the furfural reduction intermediates have readily been seen before the detection of any products by Li and Kornienko using the *operando* Raman spectroscopy.^32^

We argue that the lower onset overpotential for furfural reduction with respect to HER is already a strong indication for the dominance of a PCET based mechanism. Since HER on Cu is limited by the adsorption of hydrogen,^33^ and H* is also needed for an ECH mechanism in furfural reduction, it is hard to see how furfural reduction could proceed at a higher rate than HER, as we observe, if it proceeded *via* an ECH mechanism. The only scenario where an increase in furfural reduction activity involving surface hydrogenation compared to HER could be achieved would involve improved of H* adsorption in the presence of furfural intermediates, which we deem as unlikely.

The simulated partial current densities towards FAL and 2-MF in Fig. 3(c) are comparable owing to their similar SDS energetics (*cf.*Fig. 2(b)), in agreement with Cu's ability to produce both products in experiments. The green shaded area for 2-MF activity in Fig. 3(c), showing the spread in simulated current density upon including a 0.2 eV uncertainty in the activation energy, indicates the selectivity towards 2-MF could be enhanced by stabilizing the transition state for protonating FCHOH* to FCH*. We refrain from making any quantitative predictions on the selectivity of these two products, as a deviation of 0.1 eV in activation energy for the SDS already leads to >75% change in selectivity.^35,36^

The simulated current densities qualitatively agree with our experimental activities, shown in Fig. 3(d). We note that both the production of FAL and 2-MF suffer from mass transport limitations already at *ca.* −0.45 V *vs.* RHE indicating the previous experimental results obtained at similar conditions should be re-evaluated to deconvolute the intrinsic activity with mass transport of furfural, H_3_O^+^, and the products. By employing a fast-stirring reactor, we were able to obtain partial current densities for the major reduction products that are likely free from mass transport limitations at low overpotentials (*cf.*Fig. 3(d)). However, we recommend a more accurate product detection methodology should be developed and applied to report mass-transport-limitation-free activities for the facile biomass electrovalorization.

### 2.3 The rate-determining step lies beyond the first PCET for furfural reduction

From our microkinetic models, we predict Tafel slopes of 35 and 37 mV dec^−1^ for FAL and 2-MF production at low overpotentials (*cf.*Fig. 3(c)). This reflects our conclusion from the free energy analysis that the second PCET steps determine the intrinsic activity to FAL and 2-MF according to definition of the Tafel slope from Butler–Volmer theory,3
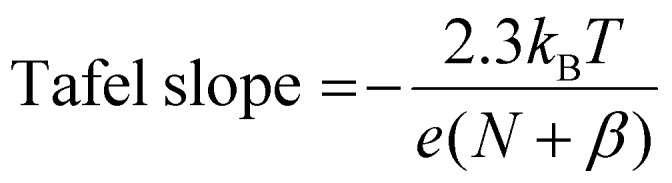
where *N* is the number of PCET steps before the RDS, *β* is the symmetry factor of the RDS, *k*_B_ is the Boltzmann constant, *T* is the reaction temperature and *e* the elementary charge.

The fitted Tafel slopes retrieved from the three lowest current densities are *ca.* 57 and 65 mV dec^−1^ for FAL and 2-MF, placing the respective RDS beyond the first protonation step in line with theory. If the first protonation were the RDS, we would expect initial Tafel slopes on the order of *ca.* 120 mV dec^−1^. Given the uncertainty in conventional Tafel slope fitting of experimental current densities,^37^ we refrain from making definite conclusions on the exact rate-determining step, but indicate that the first PCET step is unlikely to be the RDS. We also indicate the selectivity of 2-MF could benefit if the formation of FCH* from FCHOH* is promoted, *e.g.*, tuning the surface orientations to have more high-index surfaces, as suggested by the previous experiments that the roughened Cu electrodes show higher selectivity to 2-MF at *ca.* −0.4 V *vs.* RHE.^32^ The difference between experimental and theoretical Tafel slopes could result from a convolution of actual active surfaces under reaction conditions,^38,39^ varying transfer coefficient values^37^ in the electrochemical reactions and early mass transport limitations.^40^

**Fig. 4:**
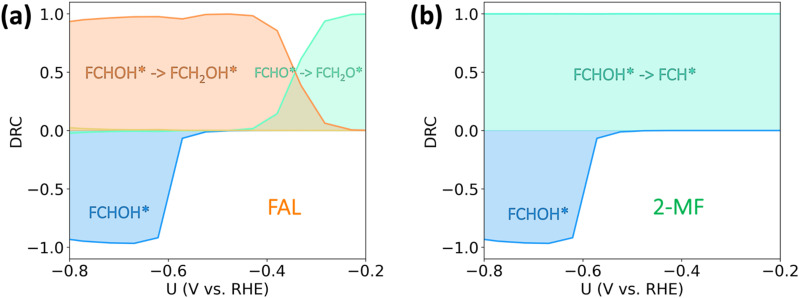
Degree of rate control (DRC) analysis of furfural electroreduction reaction on Cu. (a) and (b) are the DRC for FAL and 2-MF production respectively. Note that the number of actual reaction steps that determine the activity towards the respective products is 20. In order to increase readability, we only include the steps with DRC values > 0.1 in the plot, as the rest of the steps play a minor role in determining the overall activity.

In order to identify the rate-controlling transition states and reaction intermediates for both FAL and 2-MF within the studied potential range, we performed a degree of rate control (DRC) analysis^41^ (*cf.*Fig. 4). DRC analysis is a powerful mathematical approach that has large (positive or negative) values for the most important transition states and intermediates in the considered reaction pathway. At low overpotentials (0 to −0.2 V *vs.* RHE), the production of FAL is limited by the protonation of FCHO* to FCH_2_O* (Fig. 4(a)), suggesting at very low overpotentials (and activities), the FAL formation on Cu proceeds *via* FCH_2_O* pathway. At moderate, experimentally relevant, overpotentials (−0.2 to −0.6 V *vs.* RHE) the mechanism towards FAL changes from going *via* the thermodynamically more stable *FCH_2_O to the kinetically preferred *FCHOH (*cf.*Fig. 4(a)). In contrast, as shown in Fig. 4(b), 2-MF is limited by the protonation of FCHOH* to FCH* (DRC ≈ 1) throughout the studied potential range. Below −0.6 V *vs.* RHE, the activity towards both FAL and 2-MF reduce with an increase in the adsorption strength of FCHOH* as evidenced by a large negative DRC (inhibiting step), as the coverage of *FCHOH coverage increases and reaches saturation. In this situation, a further increase in the (already high) coverage of FCHOH reduces the coverage of all other furanic species on the surface, due to the repulsive interaction with the former. Furthermore, the saturation of the FCHOH* coverage increases the estimated Tafel slopes (*cf.* Fig. S4, ESI†). HER is determined by Volmer reaction throughout the potential range studied in this work (*cf.* Fig. S5, ESI†).

### 2.4 Electrochemical hydrogenation is unlikely to be the dominant pathway for furfural reduction on Cu

In order to better understand the role of surface hydrogenation in furfural reduction on Cu, we also simulated the ECH-based mechanisms towards FAL and 2-MF. As shown in Fig. 4(a), the formation of 2-MF and FAL are limited by the C–O bond breaking and hydrogenation of FCH_2_O* respectively. The activation free energies for these limiting steps have been determined to be 1.10 eV and 0.99 eV, respectively, which are unlikely to respond significantly to an applied potential given that no electrons are directly involved in surface hydrogenation step (*i.e.*, *β* ≈ 0. The ECH pathway *via* FCHOH* is shown in Fig. S5 (ESI†), which displays an alarmingly high activation free energy of 1.20 eV for C–O bond scission of FCHOH* to form 2-MF.

**Fig. 5:**
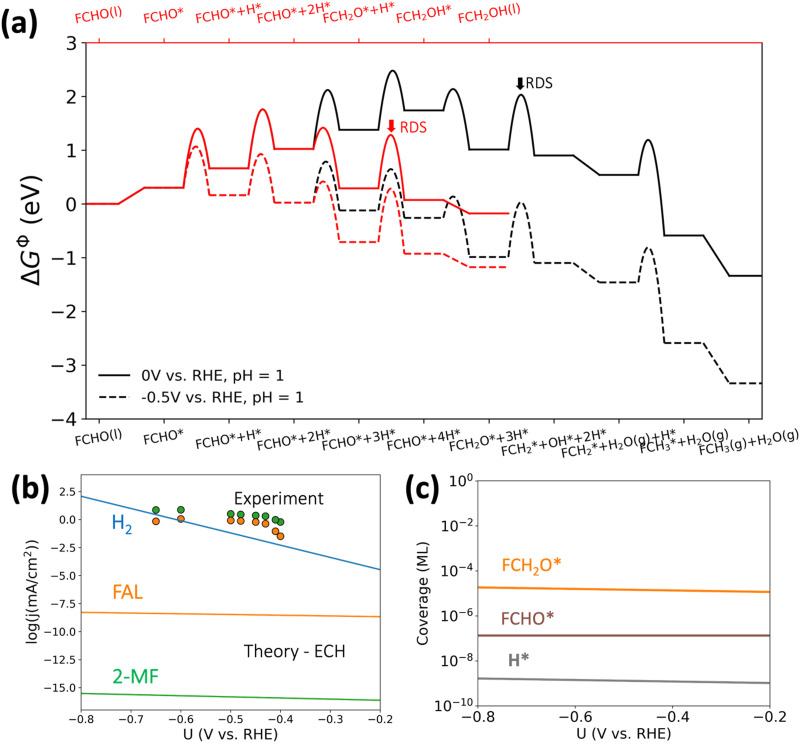
Theoretical results of furfural reduction reaction on Cu following an ECH-based mechanism. (a) The calculated constant-potential free energy diagram for furfural reduction to FAL (in red) and 2-MF (in black) at 0 and −0.5 V *vs.* RHE; (b) the simulated partial current densities (solid lines) assuming an ECH-based mechanism in comparison with experimental results (dots, *cf.*Fig. 3(d)); (c) the simulated coverages of the main surface adsorbates in the ECH-based mechanism.

The simulated current densities towards FAL and 2-MF for the ECH pathway, shown in Fig. 5(b), are more than 6 orders of magnitude lower than in the simulation based on the PCET mechanism (*cf.*Fig. 3(c)) as well as our experimental results (*cf.*Fig. 3(d)). In contrast, H_2_ is the dominant product throughout the potential region. The low activity towards FAL and 2-MF predicted for the ECH pathway originates from (i) extremely low H* coverage on Cu(111) as shown in Fig. 5(c) and (ii) high activation barriers for the surface hydrogenation of the reaction intermediates under the relevant reaction conditions.

A higher coverage of electrogenerated H* might make ECH pathway more productive, *e.g.*, on Pt and Pd electrodes. We note that Zhou *et al.* recently reported the participation of H* in furfural reduction to 2-MF on Pd-based catalysts in acid by interpreting adsorption patterns of furfural and hydrogen from *in situ* surface enhanced Raman spectroscopy (SERS).^42^ Thus, we note that the ECH mechanism might play an important role in materials where H* is formed *via* underpotential deposition or metal hydrides present under reaction conditions.^43^

The Volmer step, which in an ECH mechanism is responsible for any observed potential and pH dependence, is not predicted the rate-limiting step in furfural reduction. Thus, the Tafel slope in an ECH mechanism would be only a consequence of the coverage build-up of either H* or furanic intermediates with potential, which we show in Fig. 5(c). Once the coverages of the reactants at the RDS are saturated, the resulting current densities in an ECH mechanism would not exhibit any potential dependence (*i.e.*, *β* ≈ 0, Tafel slope ≈ ∞).

### 2.5 pH dependence of furfural reduction on Cu

We further studied the pH dependence of furfural reduction activity highlighting the competition between furfural reduction and HER in acidic conditions. The simulated rates of FAL and 2-MF against potential and acidic pH are shown in Fig. S7 (ESI†). At low overpotentials, a decrease in both potential (more positive) and pH increases 2-MF formation faster than that of FAL, because in this potential region, FAL production is limited by 1st-PCET FCHO* protonation to FCH_2_O*, while 2-MF by 2nd-PCET FCHOH* protonation (*cf.*Fig. 4), resulting in a larger response to potential/pH for 2-MF in this region. The simulated selectivity for FAL and 2-MF is presented in Fig. S8 (ESI†). To favor value-added production of 2-MF, a low pH (<1.5) and moderate potentials (*ca.* −0.5 V *vs.* SHE) is suggested in theory, which is in line with a previous viewpoint.^22^ The respective rate and selectivity for HER is then shown in Fig. S9 (ESI†). We find that HER could outcompete furfural reduction at both extremely low and high overpotentials, thus a moderate potential range is in need promote furfural reduction and suppress H_2_ evolution. The results presented above provide a theoretical rationale for selective furfural reduction by leveraging both the pH and applied potential.

**Fig. 6:**
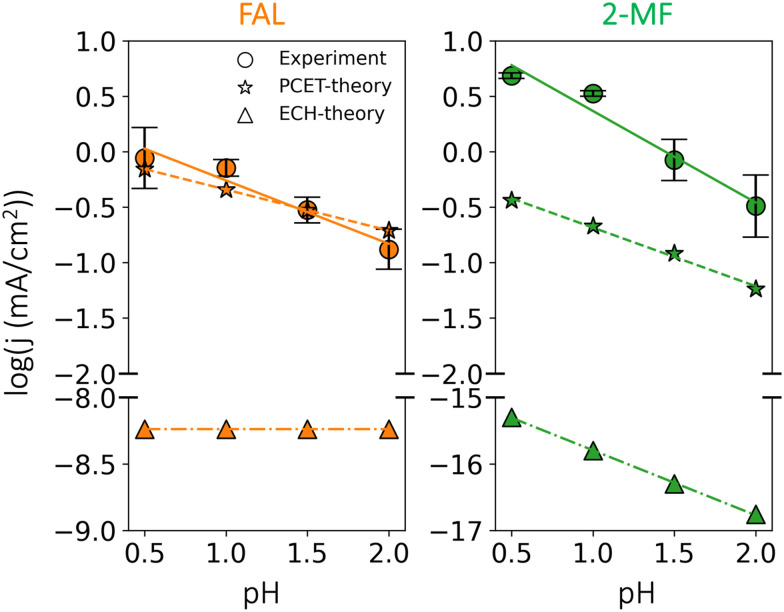
Measured and simulated partial current densities of FAL and 2-MF at varying acidic pH at −0.5 V *vs.* RHE. The solid lines represent the fitted current densities from our measurement, while the dashed and dash-dotted lines are the simulated current densities for the PCET and ECH based mechanisms, respectively. Reaction conditions: HClO_4_ electrolyte adjusted in concentration for varying pH, 8 mM furfural, potential applied was −0.50 V *vs.* RHE for 3 hours. Error bars were produced using the results of two separate experiments.

In experiments, varying the electrolyte pH between 0.5 and 2.0 at a fixed RHE potential (−0.5 V *vs.* RHE) allows us to identify the dominance of a PCET or ECH based pathway as shown in Fig. 6. Given the low electrolyte pH employed in the experiments, we can safely assume H_3_O^+^ as the dominant proton donor. A PCET based mechanism is expected to show a reduction in activity with pH in acidic conditions,^44–47^ while the ECH mechanism directly consuming surface adsorbed H* could display complex scenarios, where pH, potential and selectivity might play different roles.

As can be seen in Fig. 6, low pH favors 2-MF over FAL. The measured partial current densities towards FAL and 2-MF show a distinct negative dependence on pH: −0.57 and −0.82 dec per pH respectively. This behavior is in qualitative agreement with our microkinetic simulations of the PCET based mechanism with negative dependences of −0.37 and −0.52 dec per pH for FAL and 2-MF respectively. Quantitative differences could originate from different symmetry factors for rate-determining steps, *i.e.*, FCHOH* protonation (*cf.*Fig. 2).^44,46^ The larger pH dependence of 2-MF suggests that lowering pH could enhance the selectivity to 2-MF over FAL, in line with the experiments shown in Fig. S10 (ESI†) and previous reports.^22^

In contrast to the observations for the PCET based pathway, the simulated current density towards FAL based on the ECH mechanism is pH independent throughout the studied pH range, while that of 2-MF is strongly pH dependent (−0.98 dec per pH) as shown in Fig. 6 (ECH-theory). Nonetheless, ECH mechanism towards both products displays negligible activity that is orders of magnitude lower than simulated current densities obtained for the PCET based pathway and experiments (*cf.*Fig. 6). Therefore, in addition to the calculated energetics, the observed pH dependence on activities further strengthens our conclusion of a PCET-based mechanism to be the dominant pathway for furfural reduction on Cu electrodes.

## 3 Conclusion

In this work, we present detailed microkinetic simulations based on the constant-potential DFT energetics and pH dependent experiments to understand the reaction mechanism of furfural electroreduction on Cu. Our simulations and experiments show that Cu can produce both FAL and 2-MF in acidic conditions, where we identified the rate-determining step to lie beyond first PCET step. We then demonstrate that a surface hydrogenation-based mechanism is unlikely to be dominant for furfural electroreduction, due to the negligible H* coverage and high (potential-independent) surface hydrogenation barriers under mild reaction conditions. Measurements and simulations performed at varying electrolyte pH further strengthen the conclusions of a PCET-dominated mechanism. The mechanistic insights herein provide directions to tune the selectivity towards tailored products for furfural reduction, *e.g.*, more valuable 2-MF: (a) surface modulations to display better C–O bond breaking ability help enhance the yield and (b) low pH (<1.5) in combination with moderate potentials (*ca.* −0.5 V *vs.* SHE) favors 2-MF production. Besides, our work also sheds light on the role of surface-adsorbed H* and pH-dependent activity on Cu electrodes for multi-step electroreduction reactions.

## 4 Methods

### 4.1 Computational details

#### 4.1.1 Basic DFT calculation parameters

In this work, we applied the Solvated Jellium Method (SJM)^30^ implemented in the GPAW code^48,49^ for all the DFT calculations to consider solvation effect. An average grid spacing of 0.18 Å and a 4 × 3 × 1 *k*-points mesh were applied for the orthogonal 3 × 4 × 3 Cu(111) slab model with an ice-like water layer. Periodic boundary conditions were applied in the directions of the surface plane *i.e.*, *x* and *y* directions and open boundary conditions in the *z*-direction. At least 10 Å of vacuum/implicit solvent were applied between the atoms and the boundary in the *z*-direction. We created a field-free zone in the solvent above the asymmetric slabs *via* the dipole-correction implemented in GPAW-SJM. A Fermi smearing of 0.1 eV was used in all calculations. To better describe the interaction between metal surface and furanic molecules, we used optB88-vdW functional^50^ to account for the critical van der Waals effect on aromatic species, which has been demonstrated to relieve over-binding of the dispersion energy by PBE-D3 functionals on Cu.^51^

#### 4.1.2 GC calculation for electrochemical barriers

SJM implementation uses an effective potential cavity solvation model developed by Held and Walter.^52^ The parameters for solvation in water applied in this work:^44^ strength of the repulsion at the atomic radii controlling the cavity size *u*_0_ = 0.18 eV, surface tension 0.001148 Pa m, relative permittivity (dielectric constant) *ε* = 78.36, temperature = 298.15 K. The counter charge in SJM model was chosen as a 3 Å thick jellium slab starting two vdW-radii of oxygen atoms above the highest water molecule in the water layer. The tolerance for the electrode potential deviation from target potential was set to 5 mV in the calculation of stable reaction intermediates and 10 mV in the case of transition state searches. The reported constant potential free energies *G*^*Φ*^ (*Φ* = work function) were calculated as a sum of the constant potential energies including the constant particle DFT energy *E*^*Φ*^ = *E*^*n*_e_^ − *n*_e_*μ*_e_ at *n*_e_ excess electrons, and the electron's chemical potential *μ*_e_ = −*Φ*, the effective solvation free energy directly derived from the implicit solvation scheme inherent in SJM, and the vibrational free energy contributions (zero-point energies, heat capacity and vibrational entropy) calculated from a constant potential vibrational analysis conducted at 298.15 K.

The Nudged elastic band (NEB) method^53,54^ has been conducted to calculate the transition states (TS). For electrochemical reactions, we calculated the TS within the SJM scheme to sustain constant potential. Initial-state structures for a reduction step always have a H_3_O^+^ solvated in the single water layer, where the adsorption of furanic intermediates were sampled and the structure with the lowest energy was later applied as the initial state. To align with CHE equilibrium,^55^ which refers to a proton in bulk solution, we used the ensemble energy of the initial state of acidic NEB calculations without H_3_O^+^ and 0.5 H_2_ as the initial state energy in free energy diagrams. We calculated the PCET barriers at 0 V *vs.* SHE and pH = 1 and obtained the symmetry factor *β* by the charge transfer in GC calculations for the response of the determined electrochemical activation energies to potential. The adsorbate polarization (*γ*) is neglected due to the similar polarization of different furanic species on the surface and the negligible dipole moments of HER intermediates. As for surface hydrogenation mechanism, we only considered the Langmuir–Hinshelwood mechanism, while Eley–Rideal mechanism has been suggested unlikely on Cu electrode by May *et al.*^19^

#### 4.1.3 Microkinetic modeling

The microkinetic modeling was carried out using the CatMAP code^56^ based on the mean-field approach and the steady-state approximation including a self-consistent description of adsorbate–adsorbate interactions.^57,58^ Lateral adsorbate–adsorbate interactions were modeled using a first-order expansion in the coverage for the differential adsorption energy:
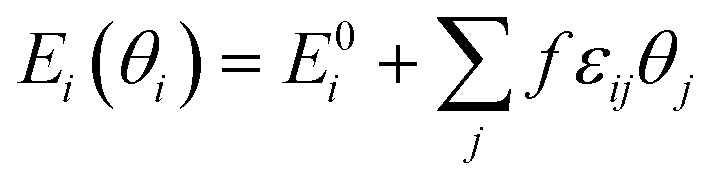
where *E*_*i*_(*θ*_*i*_) is the differential adsorption energy of species i given a vector of coverages *θ*_*i*_, *E*^0^_*i*_ is the differential adsorption energy of species *i* in the low-coverage limit, *ε*_*ij*_ is a matrix of interaction parameters for the interaction between species *i* and *j*, *f* corresponds to a piecewise-linear function for the adsorption energy as a function of coverage. As H* is much smaller than furanic species, we assume that H* barely affects the strength of interactions. All the interactions given by transition states are also neglected as a result of their low coverages by definition. Besides, the furanic species are similar in size and configurations, thus we approximated the *ε*_*ij*_ among FCHO*, FCHOH*, FCHOH*, FCH_2_O*, FCH_2_OH*, FCH*, FCH_2_* and FCH_3_* with self-interaction matrix of furfural, *i.e.*, *ε*_FCHO*,FCHO*_ = 6.82. The cross-interaction terms of different intermediates are approximated by geometric mean of self-interaction terms of two interacting adsorbates. To efficiently achieve convergence of the microkinetic models including adsorbate–adsorbate interactions, we ramped up the interaction strength from 0 to 1 with a step size of 0.1.

A Newton root-finding algorithm with a max iteration number of 500 was used to determine the steady-state rates and coverages. A decimal precision of 200 with a convergence tolerance value of 10^−50^ were used. More details could be found in ESI.†

### 4.2 Experimental details

#### 4.2.1 Electrode preparation

Cu electrodes were prepared by cutting Cu foil (99.99%, Goodfellow) into 1 × 1 cm^2^ squares and using fine sandpaper on each side to remove any external impurities. The electrodes were pierced with Cu wire which acted as the connection for the working electrode. The electrodes were then submerged in ethanol and sonicated in an ice bath for 20 min, with subsequent washing with MilliQ water before use.

#### 4.2.2 Electrochemical experiments

Electrochemical measurements were carried out in a three-electrode custom-made H-cell (Cambridge Glassware) with the anolyte and catholyte chamber separated by a Nafion 117 membrane (Fuel Cell Stores). The entire cell was boiled in ultrapure MilliQ water (18.2 MOhm) before any electrochemical experiments. The catholyte chamber was purged using Ar gas (99.998%, BOC) for 10 min before use to remove any dissolved oxygen and was not purged throughout the experiments. The working and counter electrodes were Cu foil and Au mesh respectively. We used a saturated Hg/HgSO_4_ electrode as a reference electrode which was calibrated against the reversible hydrogen electrode (RHE), hence the relevant electrochemical data in this work is reported on the RHE scale. All recorded potentials were iR corrected. The acidic electrolytes used in this work consisted of a HClO_4_ (suprapure, Merck) at varying concentrations depending on the experiment, in all three compartments.

#### 4.2.3 Product analysis

Aliquots of the catholyte solution were taken after chronoamperometry experiments to quantify any furfural reduction products. FAL and hydrofuroin (minor product) were quantified by high-performance liquid chromatography (HPLC, Agilent Infinity 1260 II) using purchased standards of FAL (98%, Sigma Aldrich) and furfural (99%, Sigma Aldrich) to produce calibration curves (Fig. S10 and S11, ESI†). Hydrofuroin standards were synthesized using Mg mediated homocoupling of furfural as no commercial standards were available. The full synthesis procedure is in detailed in the ESI.† A mixture of water : acetonitrile (88.7 : 11.3) was used as the mobile phase at a flow rate of 1 mL min^−1^, with the samples being fed through a Zorbax SB C-18 column (4.6 × 50 mm, 3.5 micron, Agilent Technologies). The column temperature was maintained at 35 °C throughout each measurement and the products were detected by a UV-Vis detector set at 220 nm. FAL and furfural had retention times of 1.35 and 1.62 min respectively, whereas hydrofuroin produced two peaks at 1.90 and 4.20 min which accounted for the two isomers formed. For simplicity, both isomers reported are combined. 2-MF was not visible in the chromatograms hence was quantified using nuclear magnetic resonance (NMR, 400 MHz Bruker AV400) spectroscopy using dimethylsulfoxide (DMSO) as an internal standard. Faradaic efficiencies (FEs) were then calculated using the following equation, where the Faraday constant is 96 485 C mol^−1^. Calibrations for product analysis are shown in Fig. S11 and S12 (ESI†).
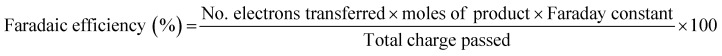


## Data availability

The data that underpin the findings of this study are available within the article and its ESI.† All computational raw data are available on GitHub https://github.com/CatTheoryDTU/furfural_electroreduction_copper.

## Author contributions

S. L., G. K., N. G. and K. C. conceptualized the work; S. L. and N. G. carried out constant-potential DFT simulations and S. L. performed the microkinetic simulations; Z. M. designed and carried out all the electrochemical experiments, electrode preparations and product analysis; S. Ch. S. helped produce the method for HPLC analysis with Z. M.; S. L. and Z. M. wrote the original manuscript; K. C., N. G., G. K., S. B. S., M. M. T., and I. E. L. S. contributed in supervision; K. C. contributed in funding acquisition for the computational work; all authors contributed in writing, review and editing.

## Conflicts of interest

There are no conflicts to declare.

## Supplementary Material

EY-001-D3EY00040K-s001
