# Supplementary material for: Unraveling the reaction mechanisms for furfural electroreduction on copper
Source: EES Catal. 2023 Apr 28;1(4):539–51. doi: 10.1039/d3ey00040k (PMC10323714; doi:10.1039/d3ey00040k)
Supplement: EY-001-D3EY00040K-s001 [file EY-001-D3EY00040K-s001.pdf]

Electronic Supplementary Material (ESI) for EES Catalysis.
This journal is © The Royal Society of Chemistry 2023

Supporting Information: Unraveling the reaction mechanisms for furfural electroreduction on copper

Sihang Liu[1,=], Zamaan Mukadam[2,=], Soren B. Scott[2], Saurav Ch. Sarma[3], Maria-Magdalena Titirici[3,4], Karen Chan[1], Nitish Govindarajan[1,5]*, Ifan E. L. Stephens[2]*, Georg Kastlunger[1]*

[1]Catalysis Theory Center, Department of Physics, Technical University of Denmark (DTU), 2800 Kgs. Lyngby, Denmark.

[2]Department of Materials, Royal School of Mines, Imperial College London, London SW27 AZ, England, UK.

[3]Department of Chemical Engineering, Imperial College London, London SW7 2AZ, England, U.K.

[4]Advanced Institute for Materials Research (WPI-AIMR), Tohoku University, Sendai, Miyagi 980-8577, Japan.

[5]Materials Science Division, Lawrence Livermore National Laboratory, Livermore, California 94550, United States.

[=]These authors contributed equally.

Email(s): govindarajan1@llnl.gov, i.stephens@imperial.ac.uk, geokast@dtu.dk

1. Supplementary methods

1.1 DFT calculations

As for closed-shell species, the calculated electronic energies are corrected using standard ideal-gas methods. The chemical potential is given by:

$$\mu = E_{elec} + ZPE + \int C_p dT - T \times S \text{ (S1)}$$

where the E_{elec} is the calculated electronic energy, ZPE the zero-point energy, C_p the heat capacity, S the entropy, and T the temperature which was assumed to be 298.15K for the furfural reduction. The free energy of liquid furfural and water was determined using the ideal gas approximation at its vapor pressure at 298.15K, ca. 0.001 bar and 0.035 bar respectively, while the gas phase H_2 is set as 1 bar. For the solvated protons, adsorbed intermediates, and transition states of the reactions, the harmonic approximation was applied to determine the free energy corrections. The reaction intermediates in the free energy diagrams are referenced to the free energy of H_2(g), H_2O(l) and furfural(l) (FCHO).

The grand-canonical formation energy of any states $\Delta G_{S*}^{\Phi}(\Phi, pH)$ in the furfural reduction reaction could then be calculated as[1]:

$$\Delta G_{S*}^{\Phi}(\Phi, pH) = G_{S*}^{\Phi}(\Phi) - G_{*}^{\Phi}(\Phi) - \sum_{i\epsilon\{C,H,O\}} n_i G_i + N_H(\Phi - 4.4 \text{ eV} + 0.059\text{pH}) \text{ (S2)}$$

where $G_{S*}^{\Phi}(\Phi)$, $G_{*}^{\Phi}(\Phi)$ and $\sum_{i\epsilon\{C,H,O\}} n_i G_i$ represent the constant potential free energies of reaction intermediates including transition states, the bare slabs and the gas(liquid)-phase references (i.e., furfural(l), H_2(g) and H_2O(l)). N_H is the number of protons transferred to form the intermediate S*. The term $(\Phi - 4.4 \text{ eV})$ represents the potential on the SHE scale.

1.2 Microkinetic modeling

The microkinetic modeling includes the elementary steps of both PCET and surface hydrogenation (SH), which are listed as follows:

1. $H^+ + e^- + * \leftrightarrow H^*$ (Volmer)

2. $H^+ + e^- + H^* \leftrightarrow H_2(g) + *$ (Heyrovsky)

3. $2H^* \leftrightarrow H_2(g) + 2*$ (Tafel)

4. $FCHO(l) + * \leftrightarrow FCHO^*$

5. $FCHO^* + H^+ + e^- \leftrightarrow FCHOH^*$

6. $FCHO^* + H^* \leftrightarrow FCHOH^* + *$

7. $FCHO^* + H^+ + e^- \leftrightarrow FCH_2O^*$

8. $FCHO^* + H^* \leftrightarrow FCH_2O^* + *$

9. $FCHOH^* + H^+ + e^- \leftrightarrow FCH_2OH^*$

10. $FCHOH^* + H^* \leftrightarrow FCH_2OH^*$

11. $FCH_2O^* + H^+ + e^- \leftrightarrow FCH_2OH^*$

12. $FCH_2O^* + H^* \leftrightarrow FCH_2OH^*$

13. $FCH_2OH^* \leftrightarrow FCH_2OH(l) + *$

14. $FCHOH^* + H^+ + e^- \leftrightarrow FCH^* + H_2O(g)$

15. $FCHOH^* + H^* \leftrightarrow FCH^* + H_2O(g)$

16. $FCH_2O^* + H^+ + e^- + * \leftrightarrow FCH_2{}^* + OH^*$

17. $FCH_2O^* + H^* \leftrightarrow FCH_2{}^* + OH^*$

18. $FCH_2OH^* + H^+ + e^- + * \leftrightarrow FCH_2{}^* + H_2O(g)$

19. $FCH_2OH^* + H^* \leftrightarrow FCH_2{}^* + H_2O(g)$

20. $FCH^* + H^+ + e^- \leftrightarrow FCH_2{}^*$

21. $FCH^* + H^* \leftrightarrow FCH_2{}^* + *$

22. $FCH_2{}^* + H^+ + e^- \leftrightarrow FCH_3{}^*$

23. $FCH_2{}^* + H^* \leftrightarrow FCH_3{}^* + *$

24. $FCH_3{}^* \leftrightarrow FCH_3(g) + *$

25. $OH^* + H^+ + e^- \leftrightarrow H_2O(g) + *$

26. $OH^* + H^* \leftrightarrow H_2O\ (g) + *$

where * represents an available surface site. All the steps have prefactors of 10^{13} based on harmonic transition state theory. Note that we simulate the electrochemical reaction in very acidic conditions (pH = 1), so we assume the OH* protonation is facile. As for the pH effect

evaluation, we applied pH and voltage as two descriptors in the microkinetic model on SHE scale and sliced the simulated production rate map at -0.5 V vs RHE.

The degree of rate control embedded in CatMap[2,3] was applied after the microkinetic simulation to understanding the determining elementary steps for different products, i.e., FAL, 2-MF and H_2. The rate control matrix is defined as:

$$X_{ij} = \frac{dlog(r_i)}{d(\frac{-G_j}{kT})} \quad (S3)$$

where r_i is the rate of production for product *i*, G_j is the free energy of species *j*, k is Boltzmann's constant, and T is the temperature, set as 298.15 K in this work.

1.3 Synthesis of Hydrofuroin

Hydrofuroin standards were synthesized using Mg mediated homocoupling adapted from Zhang et al.[4] Furfural (200 mg, 1.89 mmol) was added to 1 g of Mg turnings and left to stir overnight in a solution of 0.1 M ammonium chloride. After quenching the reaction mixture with 3 M HCl, the mixture was extracted using ethyl acetate. The organic phase was extracted further with saturated NaCl and $NaHCO_3$ to yield the crude hydrofuroin after rotary evaporation of the ethyl acetate as a brown oil. The crude product was purified using column chromatography using a gradient of *n*-hexane and ethyl acetate to yield the pure hydrofuroin. A 1H NMR spectra was taken in $CDCl_3$ to confirm the successful synthesis of hydrofuroin. Both isomers are present in the spectra and are combined for simplicity in further experiments.

2. Supplementary tables

Table S1. Major products for furfural electroreduction on metal electrodes reported in previous experiments. FAL, MF, HF and THFA represent furfuryl alcohol, 2-methyl furan, hydrofuroin and tetrahydrofurfuryl alcohol respectively.

Catalyst [Ref]	Potential (V vs RHE)	Total current density (mA/cm^2)	Selectivity (%)				Furfural Concentration (M)	Electrolyte
			FAL	MF	HF	THFA		
Al[5]	-	10	-	-	83	-	0.05	0.5 M H_2SO_4 and water–acetonitrile mixture
Ag[6]	-0.5	-	65	-	-	-	0.1	0.1 M sodium phosphate buffer at pH 6.8
Au[7]	-	75	35	-	-	-	0.25	1 M H_2SO_4
Cd[8]	-	10	-	-	43	-	0.005	10% KH_2PO_4
Cu[5]	-	10	10	80	-	-	0.05	0.5 M H_2SO_4 and water–acetonitrile mixture
Hg[8]	-	23	-	27	-	-	0.005	10% KH_2PO_4
Ni[5]	-	10	32	28	12	-	0.05	0.5 M H_2SO_4 and water–acetonitrile mixture
Pb[5]	-	10	20	-	60	-	0.05	0.5 M H_2SO_4 and water–acetonitrile mixture
Pd[9]	-0.5	-	55	-	-	-	0.1	0.1 M sodium phosphate buffer at pH 6.8
Pd[7]	-	150	20	-	-	10	0.25	n-Butanol
Pt[10]	-0.06	5-10	99	-	-	-	0.08	0.1 M H_2SO_4
Ti[11]	-	50	60	-	-	-	0.1	DMF (with 0.1% water)
Zn[8]	-	10	-	-	20	-	0.005	10% KH_2PO_4

Table S2. The calculated constant-potential activation energies at Φ=4.4 V, pH=1 and symmetry factors.

**Elementary steps**	$G_a^{\Phi}(\Phi = 4.4\ \text{V}, pH = 1)$ **(eV)**	β **(eV/V)**
$H^+ + e^- + * \leftrightarrow H^*$	1.05	0.65
$H^+ + e^- + H^* \leftrightarrow H_2 + *$	0.50	0.63
$2H^* \leftrightarrow H_2 + 2*$	0.78	0
$FCHO^* + H^+ + e^- \leftrightarrow FCHOH^*$	0.48	0.55
$FCHO^* + H^+ + e^- \leftrightarrow FCH_2O^*$	0.90	0.49
$FCHOH^* + H^+ + e^- \leftrightarrow FCH_2OH^*$	0.97	0.69
$FCH_2O^* + H^+ + e^- \leftrightarrow FCH_2OH^*$	0.98	0.30
$FCHOH^* + H^+ + e^- \leftrightarrow FCH^* + H_2O$	1.01	0.59
$FCH_2O^* + H^+ + e^- + * \leftrightarrow FCH_2{}^* + OH^*$	1.83	0.42
$FCH_2OH^* + H^+ + e^- + * \leftrightarrow FCH_2{}^* + H_2O$	1.00	0.65
$FCH^* + H^+ + e^- \leftrightarrow FCH_2{}^*$	0.60	0.65
$FCH_2{}^* + H^+ + e^- \leftrightarrow FCH_3{}^*$	0.72	0.50
$OH^* + H^+ + e^- \leftrightarrow H_2O + *$	0	0
$FCHO^* + H^* \leftrightarrow FCHOH^* + *$	0.47	0
$FCHO^* + H^* \leftrightarrow FCH_2O^* + *$	0.35	0
$FCHOH^* + H^* \leftrightarrow FCH_2OH^*$	0.25	0
$FCH_2O^* + H^* \leftrightarrow FCH_2OH^*$	0.99	0
$FCHOH^* + H^* \leftrightarrow FCH^* + H_2O$	1.10	0
$FCH_2O^* + H^* \leftrightarrow FCH_2{}^* + OH^*$	0.97	0
$FCH_2OH^* + H^* \leftrightarrow FCH_2{}^* + H_2O$	1.46	0
$FCH^* + H^* \leftrightarrow FCH_2{}^* + *$	0.42	0
$FCH_2{}^* + H^* \leftrightarrow FCH_3{}^* + *$	0.67	0
$OH^* + H^* \leftrightarrow H_2O + *$	1.11	0

3. Supplementary figures

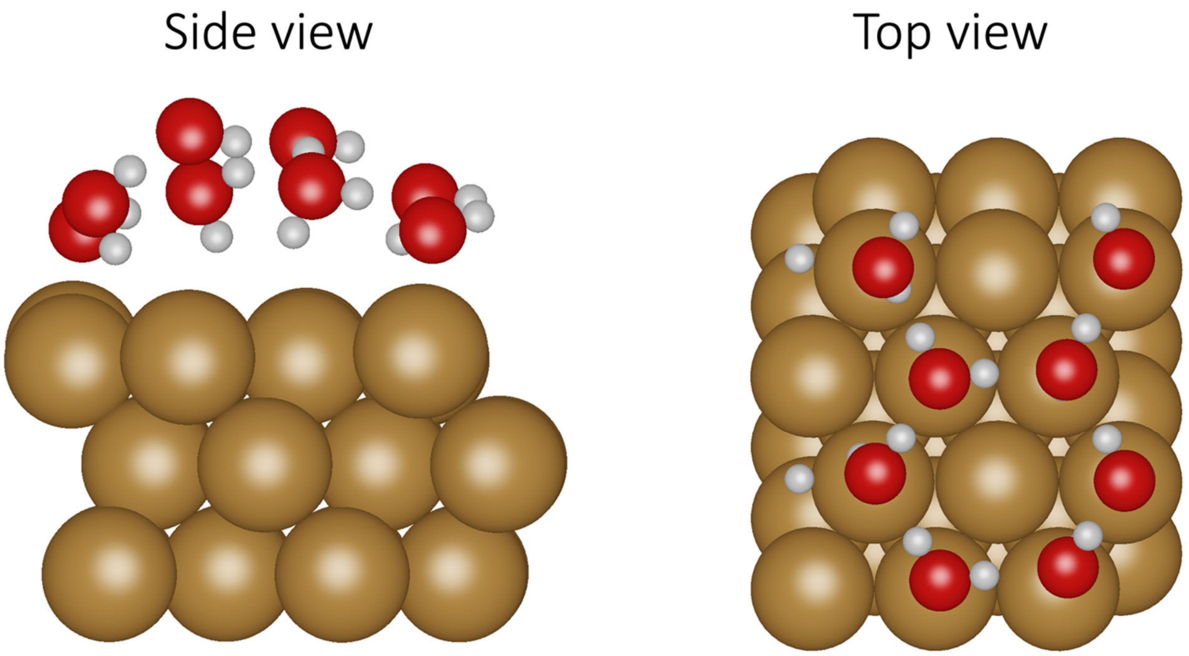

Figure S1. The computational model of Cu(111) with an explicit water layer.

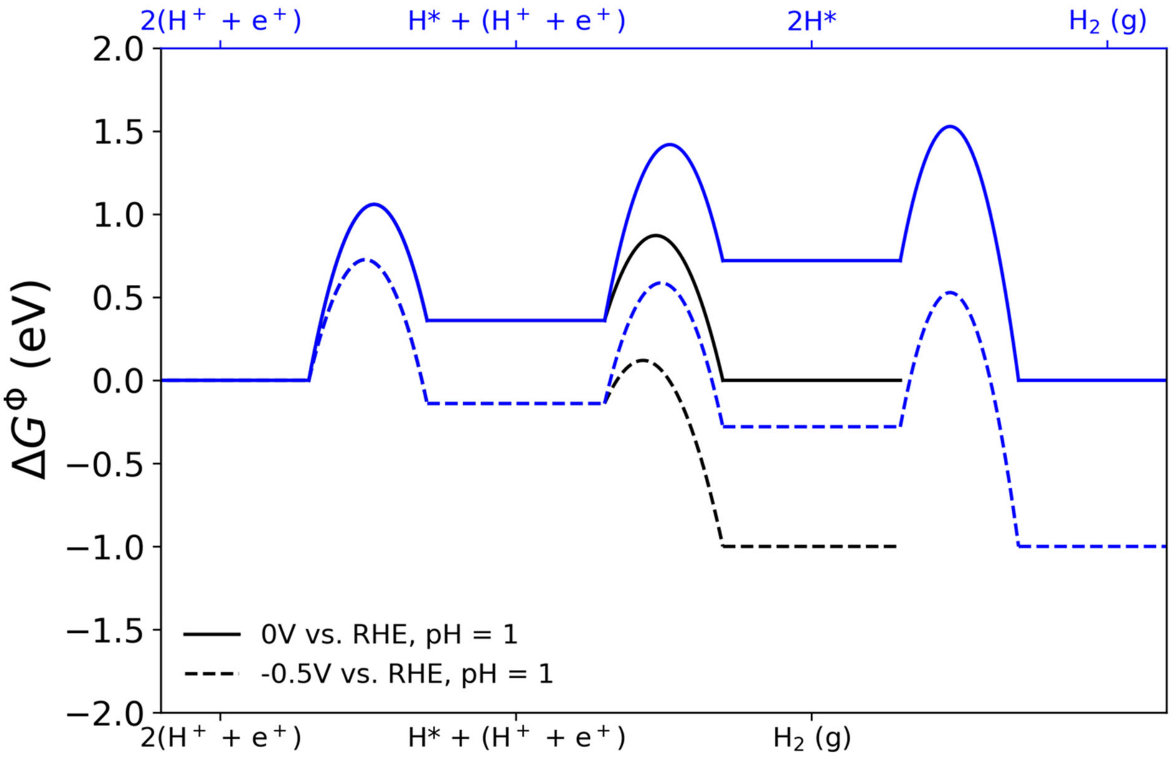

Figure S2. The calculated free energy diagram of HER on Cu(111). The black and light blue paths represent Volmer-Heyrovsky and Volmer-Tafel mechanisms respectively. Note that the Volmer-Heyrovsky pathway is energetically favored.

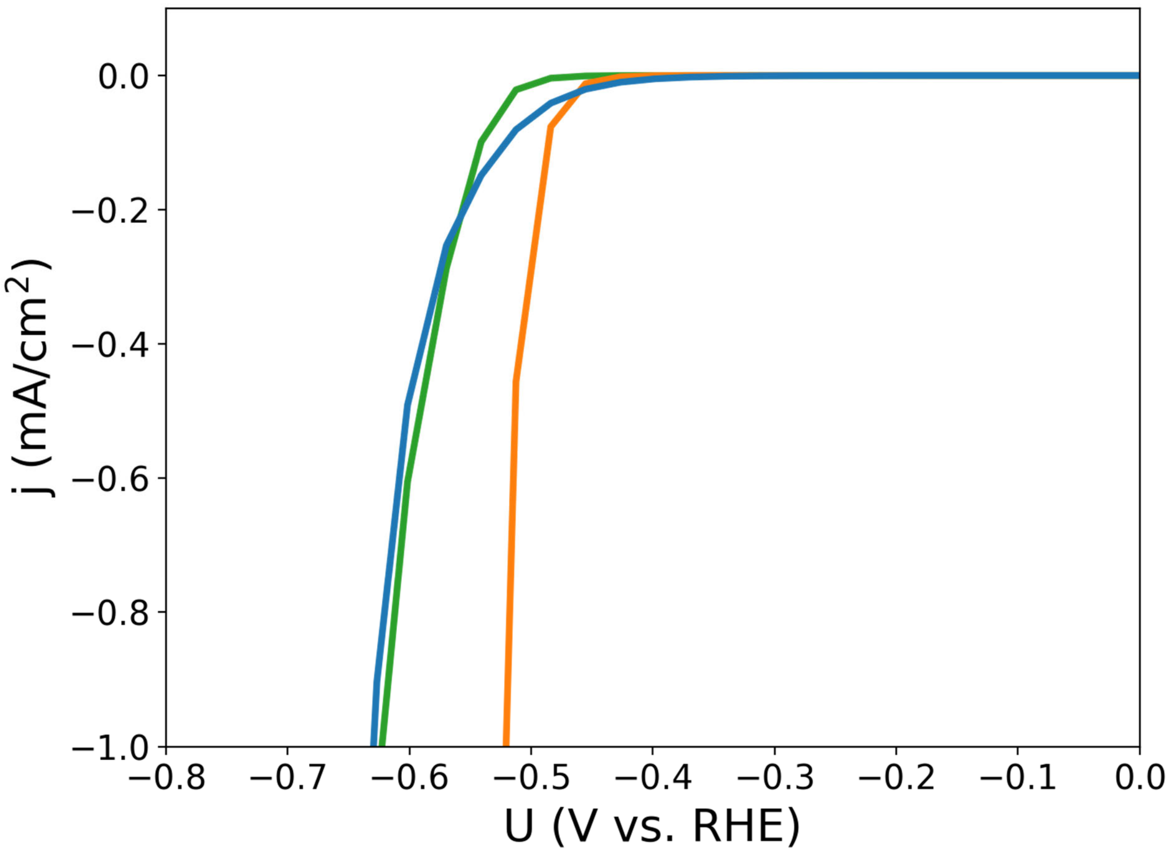

Figure S3. Simulated partial current densities of H_2 (blue), FAL (orange) and 2-MF (green) on Cu(111) at pH = 1. The partial current densities of FAL and 2-MF reach 1mA/cm^2 before the H_2.

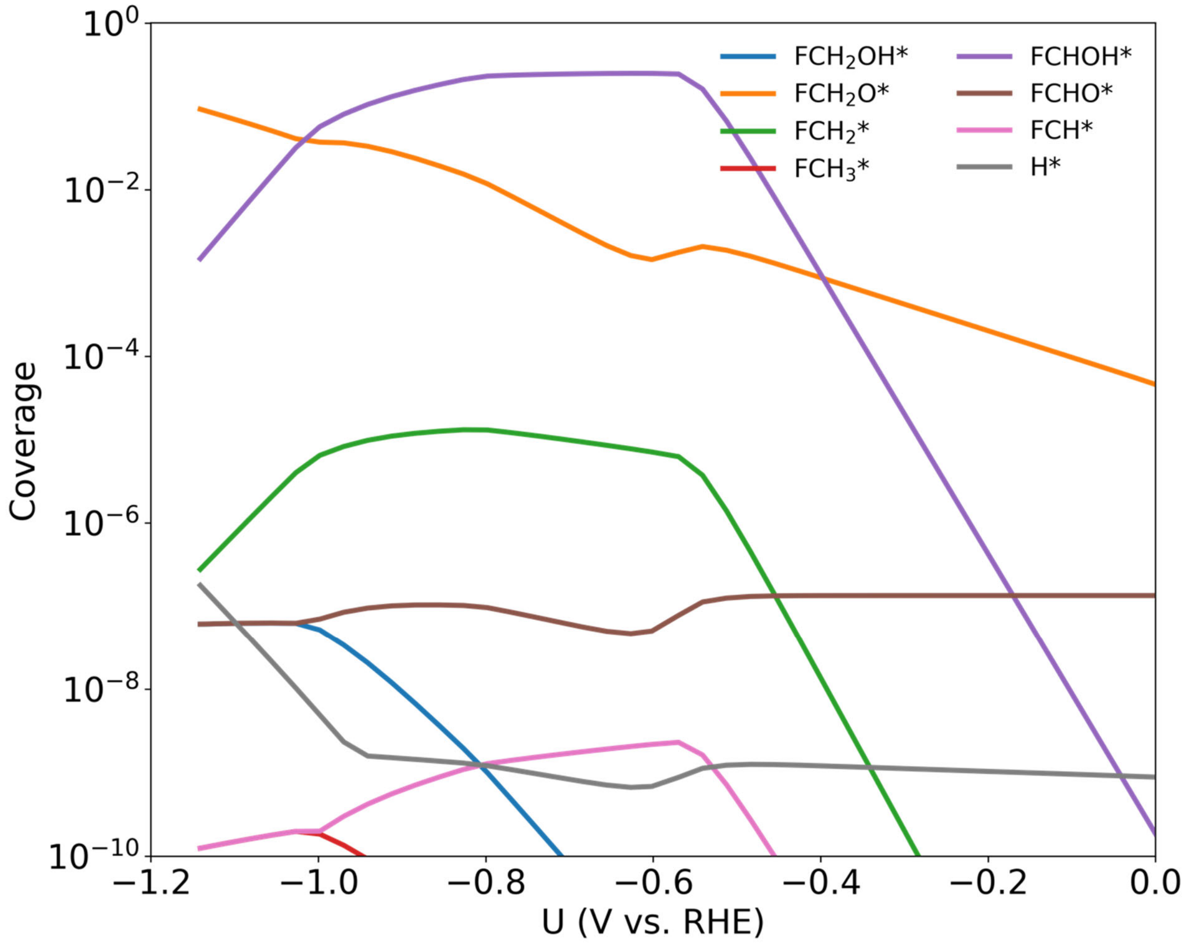

Figure S4. Simulated coverages of surface adsorbates for eFRR in PCET mechanism.

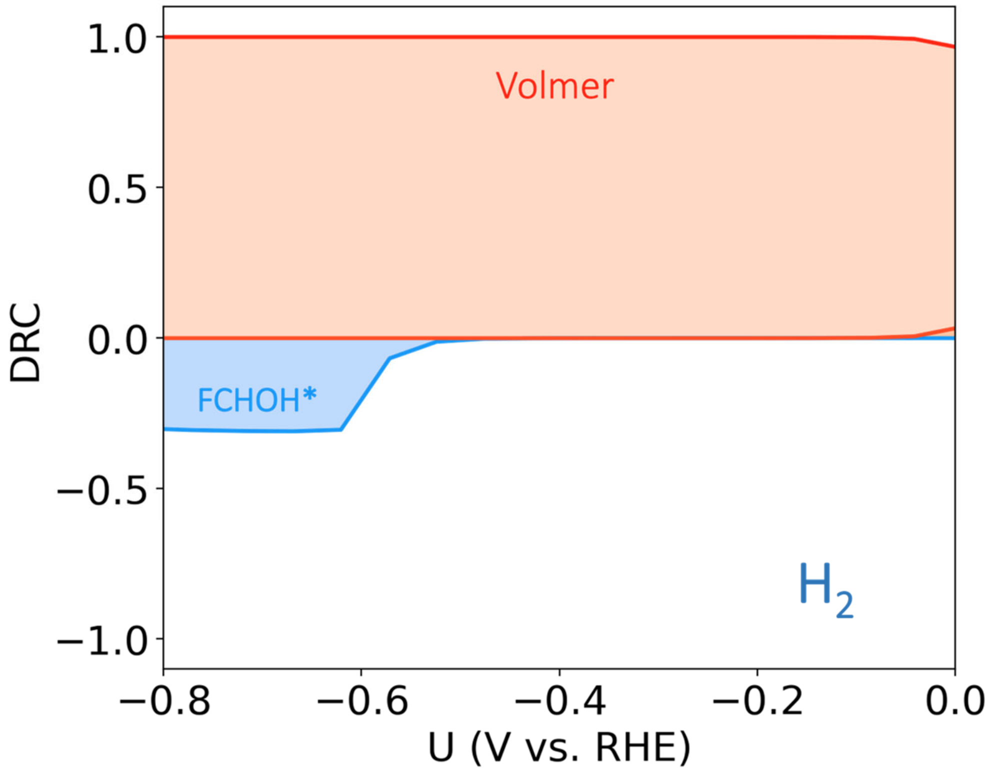

Figure S5. Degree of rate control (DRC) analysis of H_2 production during eFRR on Cu. At low and high overpotentials, HER is limited by Volmer step, while being determined by furfural reduction at moderate potentials.

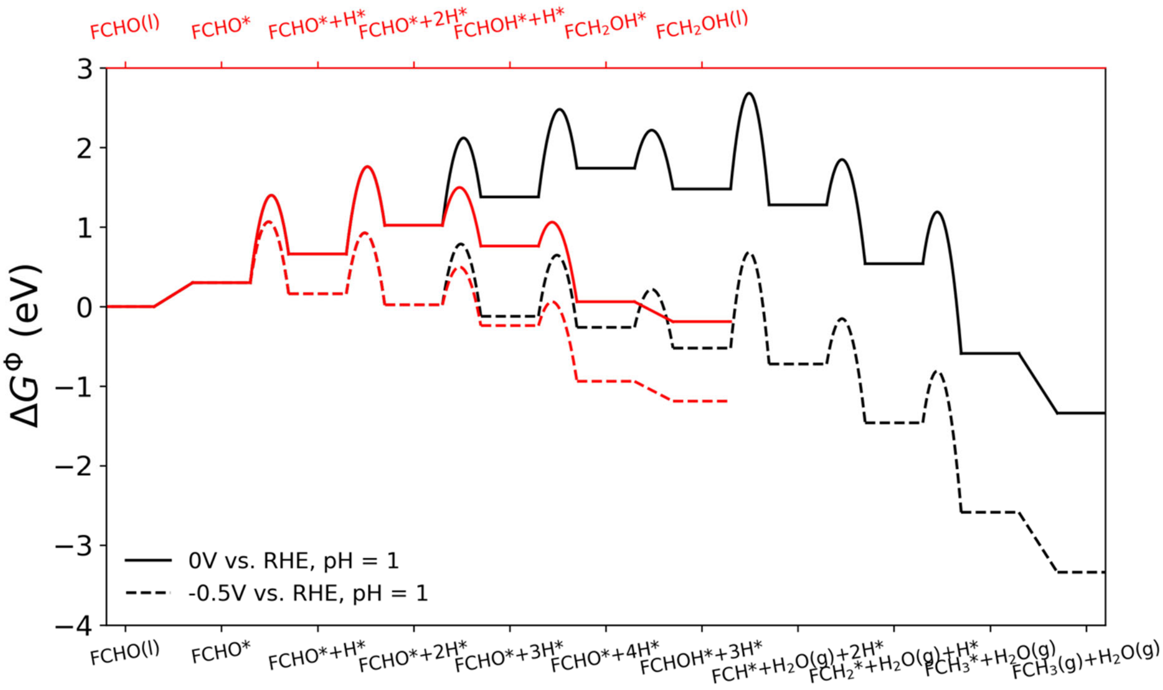

Figure S6. Free energy diagram for electrochemical furfural hydrogenation to FAL and 2-MF via FCHOH* intermediate. The RDS for 2-MF formation is FCHOH* breaking C-O bond and the RDS for FAL formation is Volmer step.

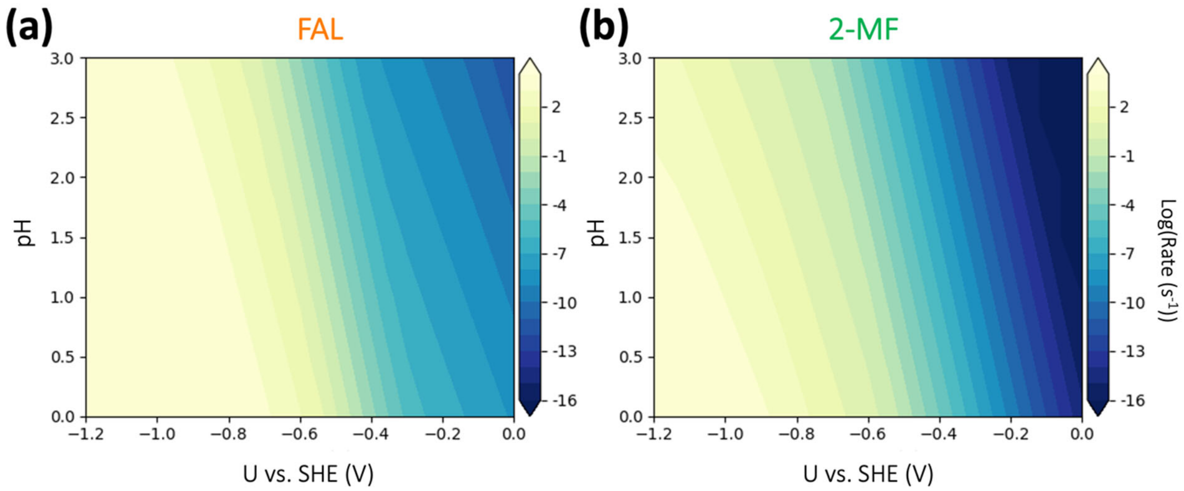

Figure S7. Simulated production rates of a) FAL and b) 2-MF at varying acidic pH (0 - 3) and potential (vs. SHE).

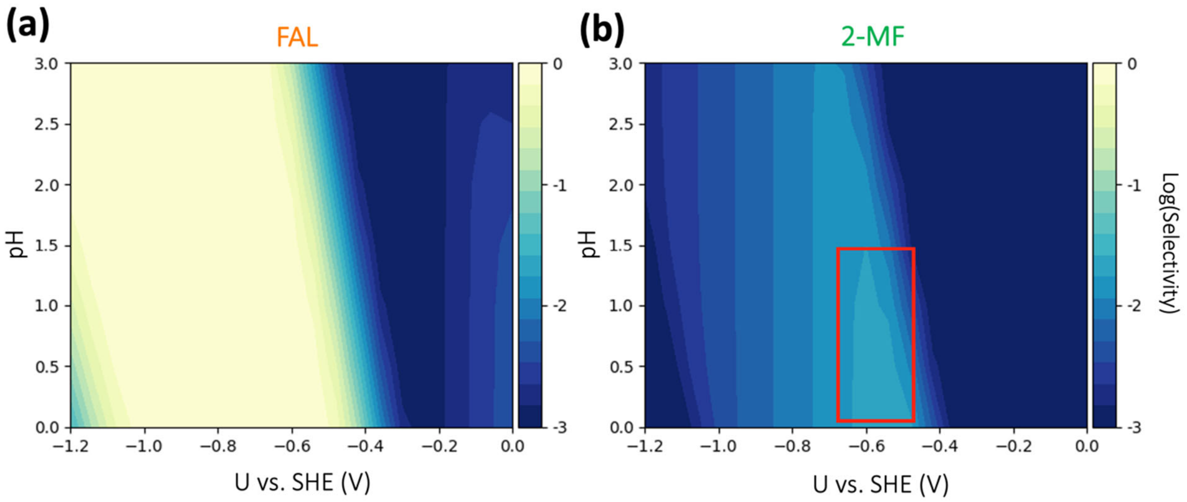

Figure S8. Simulated selectivities of (a) FAL and (b) 2-MF at varing pHs and potentials. The red box indicates the most selective reaction conditions (low pHs and potentials) for 2-MF production.

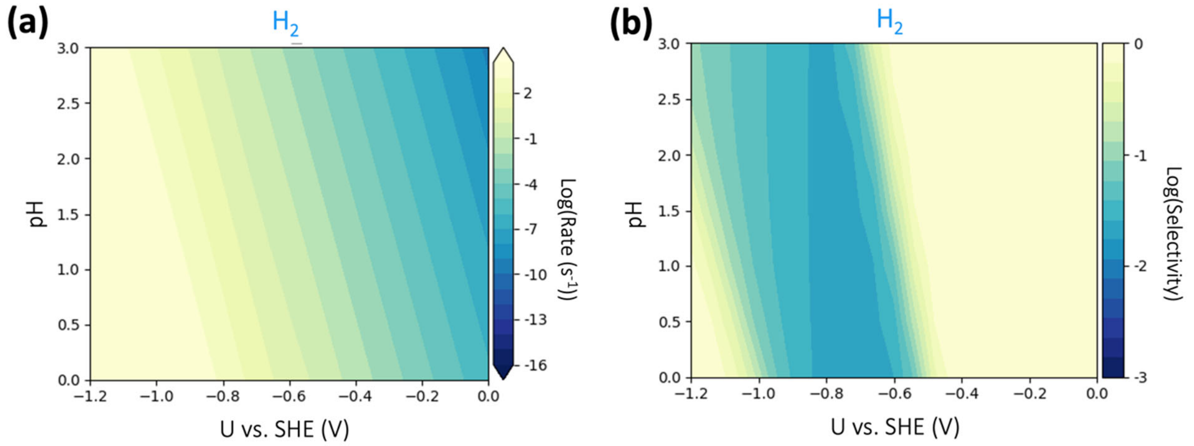

Figure S9. Simulated a) production rate and b) selectivity towards H_2 at different acidic pH (0 - 3) and potential (vs. SHE).

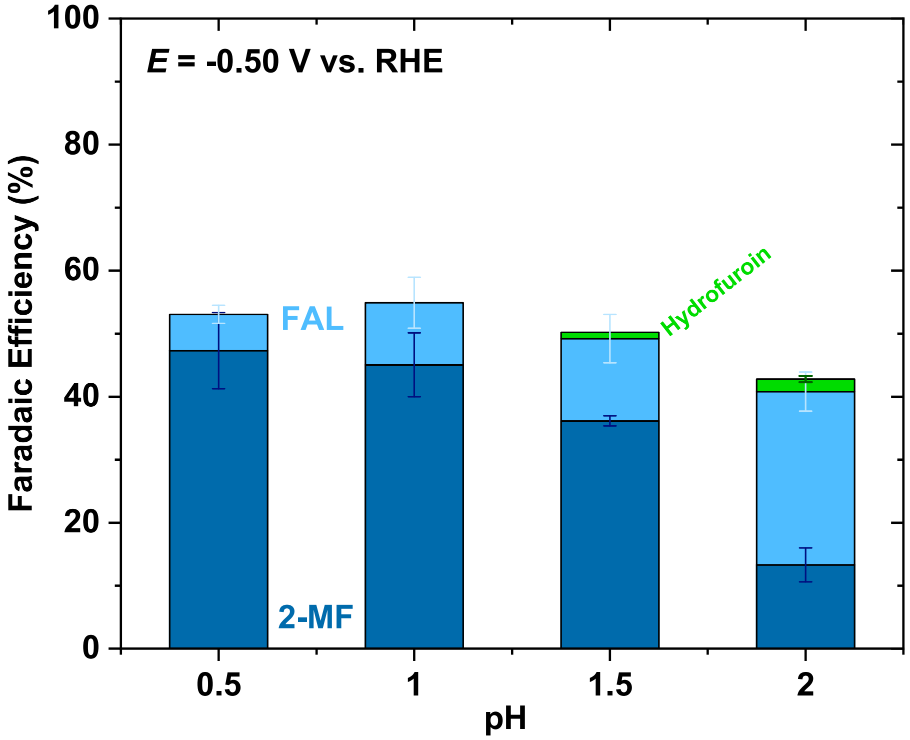

Figure S10. The experimental faradaic efficiencies of FAL, 2-MF and hydrofuroin at different pH. U = -0.5 V vs. RHE. Error bars formed from two separate experiments

Calibration Curves

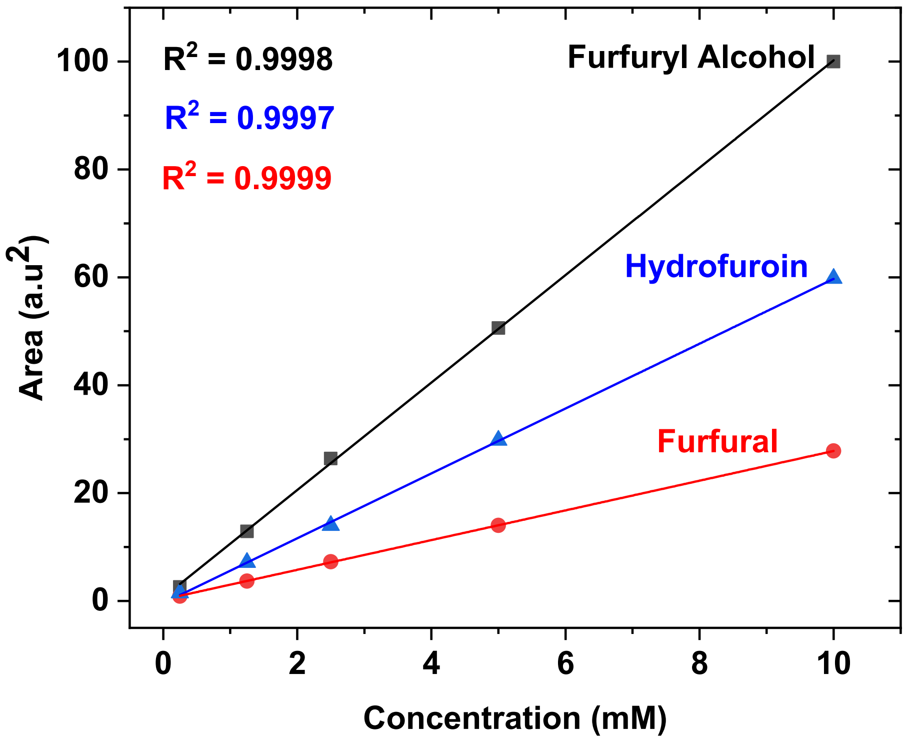

Figure S11. Calibration curves of furfural reduction products used in this study.

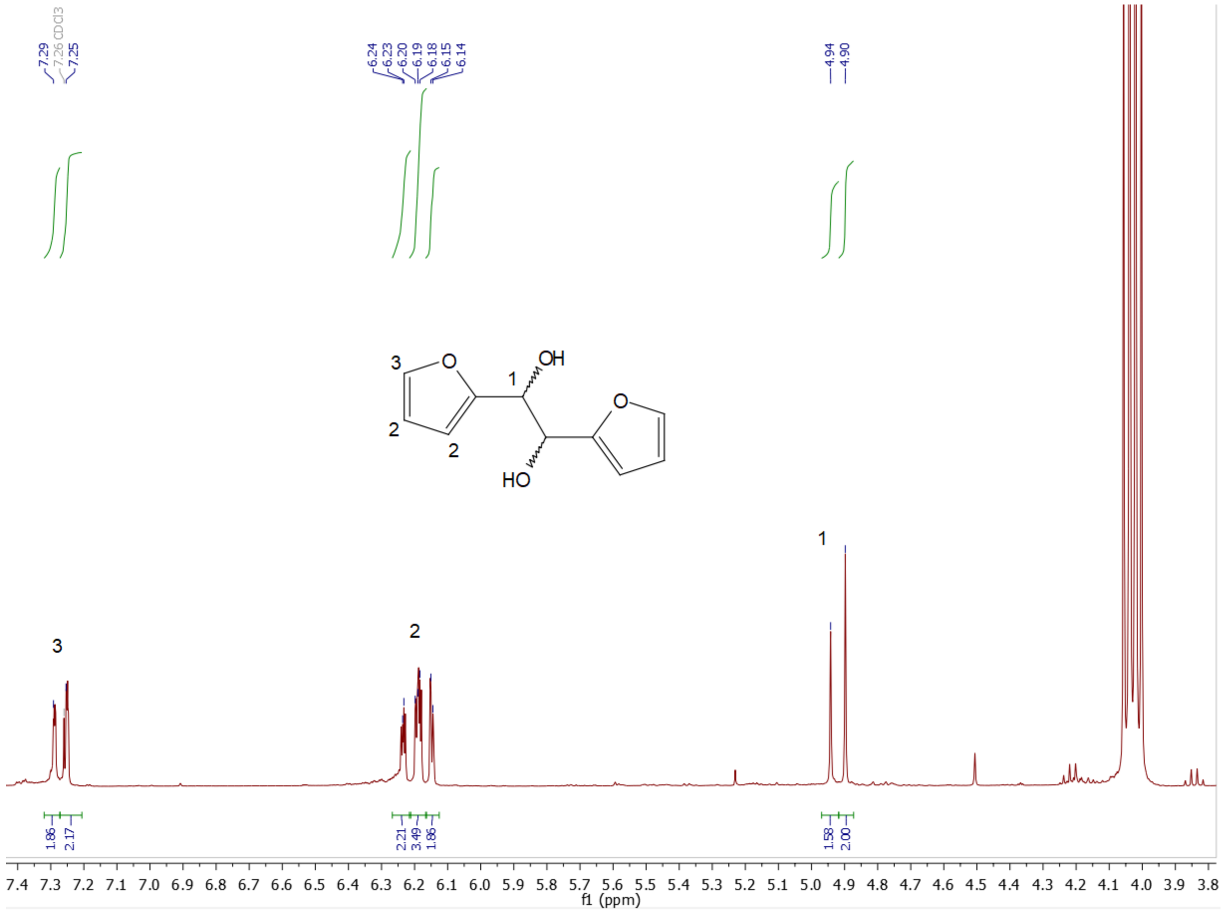

Figure S12. 400 MHz ^1H spectra of pure hydrofuroin dissolved in $CDCl_3$. Large peak at around ~4 ppm represents ethyl acetate solvent.

Supplementary references:

(1) Kastlunger, G.; Wang, L.; Govindarajan, N.; Heenen, H. H.; Ringe, S.; Jaramillo, T.; Hahn, C.; Chan, K. Using PH Dependence to Understand Mechanisms in Electrochemical CO Reduction. *ACS Catal.* **2022**, 4344–4357. https://doi.org/10.1021/acscatal.1c05520.

(2) Kozuch, S.; Shaik, S. A Combined Kinetic- Quantum Mechanical Model for Assessment of Catalytic Cycles: Application to Cross-Coupling and Heck Reactions. *Journal of the American Chemical Society* **2006**, *128* (10), 3355–3365.

(3) Stegelmann, C.; Andreasen, A.; Campbell, C. T. Degree of Rate Control: How Much the Energies of Intermediates and Transition States Control Rates. *Journal of the American Chemical Society* **2009**, *131* (23), 8077–8082.

(4) Zhang, W.-C.; Li, C.-J. Magnesium in Water: Simple and Effective for Pinacol-Coupling. *Journal of the Chemical Society, Perkin Transactions 1* **1998**, No. 19, 3131–3132.

(5) Nilges, P.; Schröder, U. Electrochemistry for Biofuel Generation: Production of Furans by Electrocatalytic Hydrogenation of Furfurals. *Energy Environ. Sci.* **2013**, *6* (10), 2925. https://doi.org/10.1039/c3ee41857j.

(6) Brosnahan, J. T.; Zhang, Z.; Yin, Z.; Zhang, S. Electrocatalytic Reduction of Furfural with High Selectivity to Furfuryl Alcohol Using AgPd Alloy Nanoparticles. *Nanoscale* **2021**, *13* (4), 2312–2316. https://doi.org/10.1039/D0NR07676G.

(7) Delima, R. S.; Stankovic, M. D.; MacLeod, B. P.; Fink, A. G.; Rooney, M. B.; Huang, A.; Jansonius, R. P.; Dvorak, D. J.; Berlinguette, C. P. Selective Hydrogenation of Furfural Using a Membrane Reactor. *Energy Environ. Sci.* **2022**, 10.1039.D1EE02818A. https://doi.org/10.1039/D1EE02818A.

(8) Albert, W. C.; Lowy, A. The Electrochemical Reduction of Furfural. *Trans. Electrochem. Soc.* **1939**, *75* (1), 367. https://doi.org/10.1149/1.3498392.

(9) Green, S. K.; Lee, J.; Kim, H. J.; Tompsett, G. A.; Kim, W. B.; Huber, G. W. The Electrocatalytic Hydrogenation of Furanic Compounds in a Continuous Electrocatalytic Membrane Reactor. *Green Chem.* **2013**, *15* (7), 1869. https://doi.org/10.1039/c3gc00090g.

(10) zhao, B.; Chen, M.; Guo, Q.; Fu, Y. Electrocatalytic Hydrogenation of Furfural to Furfuryl Alcohol Using Platinum Supported on Activated Carbon Fibers. *Electrochimica Acta* **2014**, *135*, 139–146. https://doi.org/10.1016/j.electacta.2014.04.164.

(11) Wang, F.; Xu, M.; Wei, L.; Wei, Y.; Hu, Y.; Fang, W.; Zhu, C. G. Fabrication of La-Doped TiO2 Film Electrode and Investigation of Its Electrocatalytic Activity for Furfural Reduction. *Electrochimica Acta* **2015**, *153*, 170–174. https://doi.org/10.1016/j.electacta.2014.11.203.
